# Serum uric acid response curves reveal optimal lysine requirement in quails

**DOI:** 10.1016/j.psj.2025.105949

**Published:** 2025-10-07

**Authors:** Mehran Mehri

**Affiliations:** Department of Animal Sciences, Faculty of Agriculture, University of Zabol; Sistan, Iran 98613-35856.

**Keywords:** Llysine requirement, Uric acid, Nonlinear models, Truncated Fourier Series, Morgan–Mercer–Flodin

## Abstract

This study evaluated the dietary lysine (Lys) requirement for growing Japanese quails (*Coturnix coturnix japonica*) using serum uric acid (UA) concentration as a metabolic indicator. A total of 375 seven-day-old quail chicks were randomly assigned to 25 pens in a completely randomized design with five dietary treatments (0.94 %, 1.09 %, 1.24 %, 1.39 %, and 1.54 % Lys) and five replicates of 15 birds each. Birds were fed experimental diets from 7 to 21 days of age, with Lys levels adjusted by replacing cornstarch in a wheat–soybean meal–corn gluten meal basal diet. Sinusoidal, Truncated Fourier Series (TFS), and Morgan–Mercer–Flodin (MMF) models were fitted using nonlinear regression to find the best dietary Lys to minimize serum UA. The fitted models revealed a U-shaped dose–response pattern, with the TFS model providing the best fit (R² = 0.993; RMSE = 0.178; AIC = 5.62) and estimating the Lys requirement for minimum serum UA at 1.42 %, corresponding to a predicted UA of 4.71 mg/dL. The Sinusoidal model yielded a similar estimate (1.40 % Lys), while the MMF model predicted a higher requirement (1.54 %). These results demonstrate that nonlinear curve fitting, particularly the TFS model, can accurately define dietary Lys needs in growing Japanese quails based on serum UA minimization.

## Introduction

Lysine (Lys) is a critical essential amino acid in poultry nutrition, serving as the primary limiting amino acid in corn- and soybean-based diets for Japanese quail (*Coturnix coturnix japonica*). The precise estimation of Lys requirements during key growth phases is vital, as deficiency impairs protein synthesis, retards growth, and negatively affects carcass quality, while oversupply may increase nitrogen excretion and reduce feed efficiency. Determining optimal Lys levels is particularly important during the rapid growth period of the second and third weeks of age, when protein accretion and muscle development are at their peak (Hasanvand et al., 2018; Mehri et al., 2015, 2013).

Recent research has used a variety of experimental and mathematical approaches to refine Lys requirement estimates for Japanese quail, including classical dose-response studies, response surface methodology, artificial neural networks (ANN), and hybrid statistical learning techniques. Estimates of dietary Lys requirements for optimal growth, feed conversion, and carcass traits in growing Japanese quail generally range from 1.08 % to 1.42 %, depending on the experimental model, diet composition, and the response variable assessed. For instance, increasing Lys levels up to 1.18 %–1.22 % has been shown to maximize body weight gain and carcass yield in growing quails, with some studies recommending slightly higher levels for maximizing breast muscle development (Mehri et al., 2015, 2014, 2016, 2013).

Advances in modeling, such as the use of hybrid artificial neural networks with uniform experimental designs, as well as the application of non-linear regression models, have improved the precision of requirement estimations by capturing complex growth and metabolic responses to dietary Lys (Mehri et al., 2014, 2016). Alongside conventional performance indicators, metabolic biomarkers—particularly serum uric acid—are gaining popularity as sensitive metrics for assessing amino acid adequacy and the physiological impact of dietary formulations.

Despite progress, Lys requirements may vary with genetic progress, management, environmental conditions, and growth stage, highlighting the need for continual reevaluation using robust modeling strategies and integrated metabolic and performance endpoints. Therefore, the present study was designed to determine the optimal dietary Lys level for growing Japanese quails aged 7–21 days, by evaluating serum uric acid responses with advanced non-linear and hybrid modeling approaches. This integrated strategy aims to provide a more holistic and precise recommendation for Lys nutrition, supporting both optimal growth and metabolic health in modern quail production systems.

## Materials and methods

### Ethics statements

The experimental procedures for animal trials were approved by the Animal Ethics Committee of the University of Zabol (Protocol No. AEUOZ-2012|UP-2020-BR).

***Bird management and experimental design***

Japanese quail chicks (*Coturnix coturnix japonica*) were provided a standard diet formulated to meet or exceed NRC (1994) nutritional guidelines from hatch until 6 days of age. A total of 375 seven-day-old quail chicks (mixed-sex), averaging 33.4 ± 1.07 g, were randomly distributed into 25 floor pens, with five treatment groups and five replicates per treatment, containing 15 birds per pen. The experimental house was maintained at 29°C during the second week and 26°C in the third week, with a relative humidity of 55 % ± 4.5. Lighting was set at 23 hours light and 1 hour dark throughout the study. A basal diet based on wheat, soybean meal, and corn gluten meal was formulated to supply adequate nutrients for growing Japanese quails according to NRC (1994), except for lysine (Table 1). l-Lysine.HCl was added at five levels by replacing cornstarch to achieve dietary lysine concentrations ranging from 0.94 % to 1.54 % in 0.15 % increments. Birds had ad libitum access to water and the experimental mash diets from 7 to 21 days of age. Prior to the feeding trial, all protein-containing ingredients of the basal and experimental diets were analyzed for crude protein (method 990.03, AOAC, 2006) and amino acid composition(method 982.30, AOAC, 2006). Feed samples underwent 24-hour hydrolysis in 6 N hydrochloric acid at 110°C under nitrogen. Methionine and cysteine were oxidized with performic acid before hydrolysis, while tryptophan was hydrolyzed using barium hydroxide. Amino acids were separated using a Waters HPLC system (Waters, Milford, MA), including a 1525 Binary HPLC pump, a 2487 dual-wavelength absorbance detector at 254 nm, Breeze software, and a Rheodyne 7725 injection valve with a 20 μL sample loop. Separation was achieved using a PicoTag column (3.9 × 150 mm, 5 μm particle size) Table 2.

**Table 1:** Composition of basal diet.

Ingredient	Amount (%)
Wheat	66.80
Soybean meal	10.64
Corn gluten meal	13.62
Cornstarch	1.664
Soybean oil	1.871
DL-Methionine	0.211
L-Lysine.HCl	0.297
L-Threonine	0.202
L-Glycine	0.178
L-Valine	0.149
L-Arginine	0.090
L-Tryptophan	0.073
L-Isoleucine	0.065
Limestone	1.259
Di-calcium phosphate	1.431
NaHCO3	0.076
KHCO3	0.606
NaCl	0.268
Mineral premix^1^	0.250
Vitamin premix^2^	0.250
Nutritional composition	
AME (kcal/kg)^3^	2900
CP (%)^4^	24.0
Lys (%)^4^	0.94
Met (%)^4^	0.61
TSAA (%)^4^	1.02
Thr (%)^4^	0.94
Arg (%)^4^	1.00
Ile (%)^4^	0.82
Trp (%)^4^	0.25
Val (%)^4^	1.00
Ca (%)^3^	0.85
P available (%)^3^	0.30
Na (%)^3^	0.16
DEB (meq/kg)^5^	300

1 Mineral premix provided per kilogram of diet: Mn (from MnSO4·H2O), 65 mg; Zn (from ZnO), 55 mg; Fe (from FeSO4·7H2O), 50 mg; Cu (from CuSO4·5H2O), 8 mg; I [from Ca (IO_3_)2·H_2_O], 1.8 mg; Se, 0.30 mg; Co (from Co_2_O_3_), 0.20 mg; Mo, 0.16 mg.

2 Vitamin premix provided per kilogram of diet: vitamin A (from vitamin A acetate), 11,500 U; cholecalciferol, 2100 U; vitamin E (from dl-α-tocopheryl acetate), 22 U; vitamin B_12_, 0.60 mg; riboflavin, 4.4 mg; nicotinamide, 40 mg; calcium pantothenate, 35 mg; menadione (from menadione dimethyl-pyrimidinol), 1.50 mg; folic acid, 0.80 mg; thiamine, 3 mg; pyridoxine, 10 mg; biotin, 1 mg; choline chloride, 560 mg; ethoxyquin, 125 mg.

3 Calculated values.

4 Analyzed values.

5 DEB: dietary electrolyte balance represents dietary Na + *K* – Cl in mEq/kg of diet.

**Table 2:** Estimated lysine requirements (R) to minimize serum uric acid using nonlinear models.

Model	R (%)	Uric acid (mg/dL)	R²	RMSE	AIC
Truncated Fourier Series	1.42	4.71	0.993	0.178	5.62
Sinusoidal	1.40	4.92	0.987	0.224	6.41
MMF	1.54	6.03	0.962	0.391	9.87

MMF: Morgan-Mercer-Flodin.

### Blood sampling

Prior to blood sampling, birds were fasted for 6 h with *ad libitum* access to fresh water, and sampling was performed between 09:00 and 11:00 h (approximately 2 h after the end of the fasting period, relative to the onset of the light cycle) to minimize circadian variation. Blood was collected by gentle restraint via the brachial, jugular, or metatarsal vein using a 25–27-gauge needle and 1-mL syringe, with up to 0.5 mL blood obtained per bird. Samples were transferred into plain microcentrifuge tubes, allowed to clot for 20–30 min at room temperature, and centrifuged at 2,500 × *g* for 10 minutes to separate serum. Serum uric acid (UA) concentration was determined using a commercial enzymatic uricase–peroxidase colorimetric assay kit (e.g., Randox, Sigma), according to the manufacturer’s instructions. Briefly, 10–20 μL serum was incubated with a reagent mixture containing uricase, peroxidase, and chromogen at 37°C for 10–20 min, and absorbance was measured at 546 nm using a spectrophotometer. Uric acid concentrations (mg/dL) were calculated from a standard curve prepared with known uric acid standards. Assay reliability was confirmed, with intra-assay and inter-assay coefficients of variation (CV) below 3 % and 5 %, respectively.

### Statistical analysis

Data were analyzed using GLM procedure and orthogonal contrasts in SAS (2002) measure the effects of dietary lysine on serum UA and identify the linear and quadratic trends, respectively. The Sinusoidal, Truncated Fourier Series (TFS), and Morgan-Mercer-Flodin (MMF) regression models were fitted to find the optimal lysine minimizing uric acid concentration in serum as follows:

### Sinusoidal model

y=a+b.cos⁡(cx+d)

where *x* is dietary lysine level; *a* is mean uric acid concentration around which oscillations occur; *b* is amplitude of fluctuation; *c* is angular frequency; and *d* is the phase shift.

*TFS Model*

y=a.cos(x+d)+b.cos(2x+d)+c.cos(3x+d)

where *x* is dietary lysine level; *a* is amplitude of fundamental frequency; *b* is amplitude of second harmonic; *c* is amplitude of third harmonic; and *d* is the common phase shift.

*MMF Model*

$$y=\;\frac{ab+\;cx^{d}}{b+\;x^{d}}$$

where *x* is dietary lysine level; *a* is the asymptotic serum uric acid at very low lysine (baseline); *c* is the asymptotic serum uric acid at very high lysine; *b* (> 0) is a scale parameter with units of *x^d^* that sets the inflection point $x_{1/2}=\;b^{1/d}$, where $y=\;\frac{a+c}{2}$; and *d* (> 0) is a dimensionless shape parameter controlling the steepness *d* = 1 giving a rectangular–hyperbola, larger *d* giving a sharper S-shape).

The accuracy of the fitted models was assessed using R^2^, root mean square error (RMSE), and Akaike information criterion (AIC), as below, where a higher R^2^, while lower RMSE and AIC, indicated the most precise model.

$$RMSE=\;\sqrt{\frac{\sum_{i=1}^{n}{\left(y_{i}-\;\hat{y_{i}}\right)}^{2}}{n}}$$

$y_{i}=observed\;value$

$\hat{y_{i}}=observed\;value$

n=observedvalue

$$AIC=n.\ln\left(\frac{RSS}{n}\right)+2k$$

$RSS=\;\sum_{i=1}^{n}{\left(y_{i}-\;\hat{y_{i}}\right)}^{2}=residual\;sum\;of\;squares$

n=numberofobservations

k=numberofestimatedparametersinthemodel(includingtheintercept)

## Result and discussion

Dietary Lys levels produced a highly significant effect on plasma UA (*F*_(4, 31)_ = 103.45, *P* < 0.0001). This model was very robust, explaining 93 % of the variance observed in UA concentrations (*R*² = 0.93). The relationship demonstrated exceptionally strong linear (*F*_(1, 31)_ = 345.97, *p* < 0.0001) and quadratic (*F*_(1, 31)_ = 20.43, *P* < 0.0001) characteristics.

**Fig. 1:**
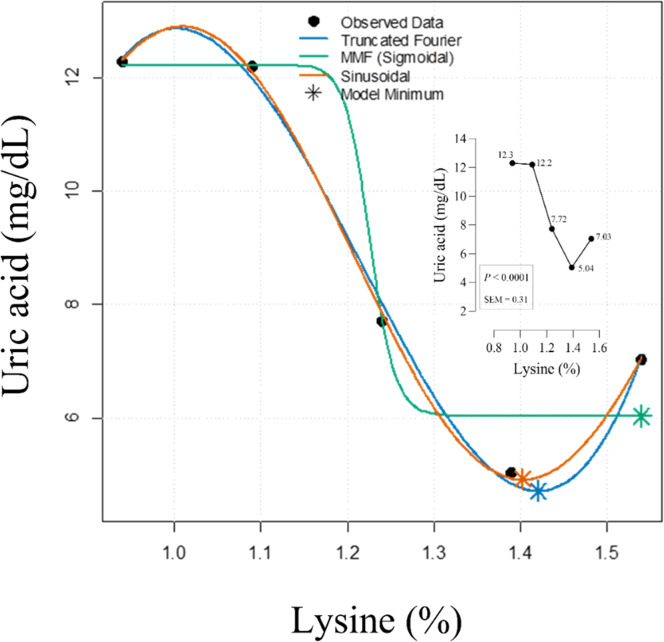
U-shaped relationship between dietary lysine and serum uric acid in growing quails, fitted with Truncated Fourier, Morgan-Mercer-Flodin (MMF), and Sinusoidal models.

The relationship between dietary Lys concentration and serum UA in growing quails exhibited a clear U-shaped pattern across all nonlinear models evaluated (Fig. 1). The TFS model estimated the Lys requirement (R) to minimize serum UA at 1.42 %, corresponding to a predicted UA concentration of 4.71 mg/dL. This model showed the best fit to the data, with the highest coefficient of determination (R² = 0.993), the lowest RMSE (0.178), and the smallest AIC (5.62) among the models tested. The Sinusoidal model produced a similar requirement estimate of 1.40 %, with a slightly higher predicted UA concentration (4.92 mg/dL) and a modest reduction in model performance (R² = 0.987; RMSE = 0.224; AIC = 6.41) compared with the TFS model. In contrast, the MMF model, a sigmoidal model, predicted a higher Lys requirement of 1.54 % and a greater minimum uric acid concentration (6.03 mg/dL). Its fit statistics (R² = 0.962; RMSE = 0.391; AIC = 9.87) indicated a weaker fit relative to the other two models. Overall, the TFS model provided the most accurate and parsimonious description of the U-shaped response, supporting its use for estimating Lys requirements in this context.

The current study's findings on the Lys requirement for growing Japanese quails, determined via nonlinear modeling of serum UA responses, align well with a growing body of research validating UA as a sensitive biomarker to assess amino acid adequacy in birds.

A seminal study on chicks fed crystalline l-amino acid diets found that UA excretion decreased and weight gain increased linearly as Lys approached the requirement, plateauing at about 0.85–0.88 % lysine, where UA levels were minimized. This reinforces our observation of a U-shaped UA response indicating that both deficiency and excess Lys provoke elevated UA excretion and metabolism. It also supports the usefulness of UA as a metabolic biomarker for efficient nitrogen utilization and amino acid balance, consistent with our modeling that centered on minimum serum UA for requirement definition (Miles et al., 1974). More recent work highlights the growing application of UA and related nitrogen metabolites as biomarkers in poultry and other species to detect amino acid imbalances and optimize diets under constant protein intake conditions. For example, studies in broilers and other livestock have confirmed plasma UA or serum urea nitrogen (SUN) as valid indicators for timely detection of limiting amino acids such as lysine and methionine, supporting more precise feed formulations (Cambra-López et al., 2022). This biomarker approach complements traditional growth performance parameters by providing early metabolic feedback on amino acid adequacy, an advantage echoed in the high accuracy of our nonlinear models used.

In comparison, the use of nonlinear models to fit nutrient-response curves has been shown to capture the complexity of metabolic responses better than traditional linear or broken-line analyses. Our finding that the TFS model provided the best fit with R² = 0.993 and the clearest U-shaped pattern parallels advances in nutritional modeling that emphasize flexible functions capable of representing both suboptimal and excessive nutrient effects on metabolic biomarkers. The inability of the sigmoidal Morgan–Mercer–Flodin model to fit this U-shaped curve as effectively highlights the importance of matching model choice with biological response characteristics (Marín-García et al., 2024). The MMF model is fundamentally a sigmoidal (S-shaped) function, designed to describe growth or saturation phenomena approaching an asymptote. Since serum UA exhibits a U-shaped response (elevated in both deficiency and excess), the MMF model was mathematically ill-suited to capture the minimum point (trough), leading to its prediction of a higher, less accurate requirement and minimum UA concentration. The TFS model's superior fit is therefore due to its mathematical flexibility in describing this complex, non-monotonic biological pattern. Similar nonlinear modeling approaches have been employed in recent poultry studies to estimate lysine requirements based on performance traits, though few have integrated metabolic biomarkers such as UA. For example, lysine requirements in growing meat quail reached levels around 1.02 % to 1.12 % for optimal protein deposition and growth, showing the biological plausibility of our UA-based estimate at 1.42 %, which may reflect the added sensitivity of metabolism-focused markers or the specific age range studied (Siqueira et al., 2021). It is important to note that growth performance data (body weight gain, feed intake, feed conversion ratio, and carcass traits) from the same experimental flock were reported previously by Hasanvand et al. (2018). In that study, the estimated lysine requirements based on performance ranged between 12.39 and 13.80 g/kg of diet, depending on the response criterion (gain, feed efficiency, breast meat yield, or dressing percentage). These values are slightly lower than the lysine requirement estimated in the present study using serum UA (1.42 % or 14.2 g/kg), which may reflect the greater sensitivity of UA as a metabolic marker compared with performance traits that often represent a later or less subtle manifestation of nutrient inadequacy. This observation supports the concept that metabolic biomarkers such as UA may identify lysine requirements that optimize metabolic status before growth performance is maximized. Nevertheless, as highlighted in previous research, performance outcomes remain the ultimate measure of dietary adequacy, and thus UA-based estimates will be interpreted in conjunction with these conventional indicators. The integrated use of nonlinear biochemical markers like serum UA combined with curve-fitting models provides a refined lens to assess amino acid nutrition, precise enough to detect subtle deviations from adequacy that can impact nitrogen metabolism and feed efficiency. This approach supports emerging trends in precision nutrition that seek to optimize diet formulations not only for growth traits but also for environmental sustainability by minimizing nitrogen excretion.

In conclusion, this study demonstrates that serum UA concentration, combined with advanced nonlinear modeling, offers a highly effective approach for defining optimal lysine requirements in growing Japanese quails. The U-shaped dose-response captured by the TFS model provides a nuanced biochemical indicator of Lys adequacy and excess, facilitating precision nutrition strategies in modern quail production. Future research integrating broader metabolic, growth, and environmental outcomes will further enhance diet formulation frameworks that support efficient, sustainable, and profitable poultry production systems.

## CRediT authorship contribution statement

**Mehran Mehri:** Writing – review & editing, Writing – original draft, Project administration, Methodology, Investigation, Formal analysis, Data curation, Conceptualization.

## Disclosures

The authors declare that they have no known competing financial interests or personal relationships that could have appeared to influence the work reported in this paper.
